# Knowledge, Attitudes, and Perception towards COVID-19 among Medical Students in Yemen: A Cross-Sectional Survey

**DOI:** 10.3390/idr14060086

**Published:** 2022-11-14

**Authors:** Ruqaiah H. Al-Ghazali, Eman S. Barhoom, Khawla A. Dahdah, Khulood S. Basalem, Tayba A. Mugibel, Khalid M. Sumaily, Essa M. Sabi, Ahmed H. Mujamammi, Saleh M. Ben Salman, Lotfi S. Bin Dahman

**Affiliations:** 1 College of Medicine, Hadhramout University, Mukalla 50511, Yemen; 2 Clinical Biochemistry Unit, Pathology Department, College of Medicine, King Saud University, Riyadh 11461, Saudi Arabia; 3 Chef Consultant of Neurosurgery, Neurosurgery Department, Johanniterkankenhaus Stendal Holunderweg University, 539576 Stendal, Germany; 4 Department of Medical Biochemistry, College of Medicine, Hadhramout University, Mukalla 50511, Yemen

**Keywords:** COVID-19, medical student, KAP, web based survey

## Abstract

Numerous measures have been taken to slow the Coronavirus disease (COVID-19) rapid spread. Such population control techniques may have a substantial impact on people’s attitudes, knowledge, and perception of COVID-19. This web-based cross-sectional survey aimed to assess Knowledge, Attitude, and Practices (KAP) towards COVID-19 among Hadhramout University Medical Students in Yemen from 15 June to 26 June 2020. This survey was performed using social media via the Google Platform among 422 Hadhramout University Medical students. After consenting, participants completed an online survey assessing sociodemographic data, 21 knowledge items, 15 attitudes items, and 5 perception items towards COVID-19. Of the total 422 participants, 389 (92.18%) were surveyed online, and 256 (65.8%) were females, and 133 (34.2%) were males aged 19-24 years (88.7%), studying medicine (58.9%), and living in urban areas (80.7%). The survey revealed that 64.0% of participants had good knowledge about the disease and 52.7% had positive attitudes towards protective measures against the virus. The majority of participants (98.2%) thought that the virus was transmitted through nasal droplets, and 59.6% agreed that the disease is dangerous. The majority of participants agreed that fever (99.2%), dry cough (97.9%), and difficulty breathing (99.5%) are the most common symptoms of the disease. The survey also showed high knowledge levels about preventive measures against the virus spreading, such as regular proper hand hygiene (99.7%), maintaining an appropriate distance (99.2%), avoiding touching eyes and nose (98.7%), and wearing facemasks in public places (97.4%). Moreover, 69.7% of participants agreed to be isolated at home if they got an infected person, 64.3% implemented washing hands with soap and water, 41.9% agreed to be separated at the hospital until they proved free from the disease, 46.0% agreed to inform the health authorities if they had any symptoms associated with the disease. By using sample T-test and analysis of variance (ANOVA), mean knowledge score about COVID-19 was significantly higher in males than in females (*p* = 0.029). Additionally, medicine students had significantly higher mean knowledge score than students of medical laboratory (*p* < 0.001) and nursing (*p* = 0.008). In general, our research revealed that participants had favorable opinions regarding the disease’s preventative measures and a good awareness of it. However, more educational initiatives and campaigns that take into account KAP modifying elements are needed.

## 1. Introduction

A rapidly contagious disease first appeared in Wuhan, China, in late December 2019 [1]. The disease was caused by a member of the family of coronaviruses, named severe acute respiratory syndrome coronavirus-2 (SARS-CoV-2). The highly contagious virus caused the disease called coronavirus disease-2019 (COVID-19) and become a global public health crisis [2]. The World Health Organization (WHO) declared COVID-19 a global pandemic on 11 March 2020 [3]. With a 5.7% fatality rate, the disease had spread to more than 200 countries [4].

The primary pathogens of the respiratory system are indeed coronaviruses. They are part of a wide family of single-stranded Ribonucleic acid (RNA) viruses that have been linked to illnesses ranging from the common cold to those with severe symptoms, like Middle East Respiratory Syndrome (MERS) and Severe Acute Respiratory Syndrome (SARS) [5]. The most common symptoms of COVID-19 are fever, dry cough, and tiredness. Other less common symptoms that may affect some patients include aches and pains, nasal congestion, headache, conjunctivitis, sore throat, diarrhea, loss of taste or smell, or a rash on skin, or discoloration of fingers or toes. Some people become infected but only have very mild symptoms [5]. Older people and those with underlying medical problems like high blood pressure, heart and lung diseases, diabetes, or cancer, are at higher risk of developing a severe illness [6].

COVID-19 transmission from human to human has been observed in health care, community, and family settings [7]. It spreads from person to person by close contact through saliva droplets or nose discharge [7]. Based on the epidemiological investigations, the incubation period of COVID-19 infection is between 1 and 14 days. Besides, the virus is contagious in asymptomatic patients [8]. Therefore, the best way to prevent this is to avoid being exposed to the infection by washing hands with soap and water frequently, using hand sanitizer, using face masks, maintaining respiratory hygiene, and maintaining social distancing [7]. Since healthcare systems in middle- and low-income countries have a limited capacity for pandemic response, public education about how to treat highly contagious respiratory disorders is crucial for preventing the spread of infection [9].

In Yemen, the first confirmed case was recorded on 10 April 2020, in Al-Shiher city, located in Hadhramout Governorate [10]. On 29 April 2020, suspected cases started in random areas of Yemen. According to the Supreme National Emergency Committee for Coronavirus, 469 confirmed cases were recorded across Yemen, with 111 related deaths [6,7]. Hadhramout was the most exposed area to infection, with 299 confirmed cases [11,12]. In June 2020, 835 new confirmed cases of COVID-19 were recorded, with 232 associated deaths, an increase from 321 cases reported in May and 79 deaths. Since the first case was reported on 10th April 2019, the authorities have recorded 1162 cases throughout Yemen, including 313 fatalities and 490 recoveries [13]. However, COVID-19, coupled with other infections like dengue, chikungunya, and malaria fevers, made the situation worse [14].

Medical students are the first people who have close contact with those infected with COVID-19. One Chinese study addressed the psychological impact of the COVID-19 epidemic on college students during the pandemic and stated that they experienced high levels of psychological stress, which may lead to undesirable effects on their education and overall psychological wellbeing [15].

Public health prevention and promotion rely heavily on knowledge, attitudes, and perception (KAP). It involves a variety of viewpoints regarding the disease’s etiology and aggravating factors, the recognition of symptoms, the available therapeutic options, and the potential outcomes [16]. Information about COVID-19 is gathered from a variety of sources, including studies of related viral diseases, governmental data, social media, the internet, prior personal experiences, and medical sources. The authenticity of these beliefs may influence various preventative behaviors and may differ throughout the population [16]. Therefore, the goal of this survey was to evaluate KAP toward COVID-19 among a convenience sample of medical students at Hadhramout University in Yemen.

## 2. Materials and Methods

### 2.1. Study Design and Population Selection

This web-based cross-sectional survey was conducted using the Google Platform Online among Hadhramout University Medical Students in Mukalla, Yemen, from 15 June to 26 June 2020. Among 422 Hadhramout University Medical Students of medicine, pharmacy, medical laboratory, and nursing colleges. Only 389 (92.18%) responded to this survey. Participants were recruited by convenience sampling following informed consent.

### 2.2. Data Collection

Data were collected using a previously designed questionnaire provided in two languages (Arabic and English) [17]. The questionnaire was distributed in Arabic, the official language in Yemen, using the Google Platform, and the link was shared with the public on social media, such as Facebook and WhatsApp. Participants were requested to fill in all items in the online questionnaire, otherwise they could not proceed to the next page. The questionnaire was divided into four parts:

1. Sociodemographic characteristics of the participants, including age, gender, residence area, educational level, and specialty.
2. Knowledge of COVID-19, including sources of COVID-19 information, COVID-19 mode of transmission, COVID-19 symptoms, and preventive measures adopted to avoid contracting COVID-19 (21 questions).
3. Attitudes and behaviors that may be associated with the prevention of the spread of the virus (15 questions).
4. Perception about the virus and the disease including the dangers of the disease, possibility that another family member is infected with the virus, the infection is associated with stigma, the media coverage of the disease, and the virus is initially designed as a biological weapon (5 questions).

The knowledge section consisted of 21 questions, and each question had a possible response of “Yes”, “No” and “Not sure”. The correct answer (Yes) was coded as 1, while the wrong answer (No/Not sure) was coded as 0. The total score ranged from 0 to 5, with an overall greater score indicating more accurate knowledge. The attitude section consisted of 15 questions, and the response to each question was indicated on a 5-point Likert scale as follows 0 (“Don’t agree”), 1 (“Not sure”), 2 (“Agree”), 3 (“Strongly agree”), and 4 (“Do strongly agree”). The total score was calculated by summating the raw scores of the 15 questions, indicating more positive attitudes towards COVID-19. The perception section included 5 questions, and each question had a possible response of “Yes”, “No” and “Not sure”. The correct answer (Yes) was coded as 1, while the wrong answer (No/Not sure) was coded as 0. The total score ranged from 0 to 5, with an overall greater score indicating more frequent practices towards COVID-19.

### 2.3. Validation and Trialling of the Study

A preliminary stage was conducted to assess the validity and reliability of the previously designed questionnaire [17]. Initially, the questions were modified to be convenient for the participants, then reviewed by experts to assess the degree to which items in the questionnaire are relevant and correctly measure knowledge, attitudes, and perception of the participants towards COVID-19. Thus, we conducted a trialing of questionnaires on 18 participants from the target population who were excluded later from the study sample. Data were used to assess internal consistency reliability using Cronbach’s alpha as well as test-retest reliability using the intra-class correlation coefficient. The results showed adequate internal consistency reliability.

### 2.4. Sampling

The sample was selected by the conventional method. The estimated sample size of n = 422 participants in this study was calculated using the WHO formula [18]. The probability of having good knowledge was obtained from a similar survey conducted on the Egyptian population [16].

### 2.5. Ethical Considerations

The study protocol followed the Declaration of Helsinki and was approved by the Ethics Committee of the Medicine College, Hadhramout University, Yemen (Ethical Approval number: CM/REC/07/2020). Participants who agreed opened the sent Google form link and filled it in, with their right to continue or withdraw.

### 2.6. Statistical Analysis

Descriptive analysis was used to summarize sociodemographic data and responses to questions concerning knowledge, attitudes, and perception towards COVID-19. Data were summarized as frequencies (n) and percentages (%) for categorical variables. The mean knowledge score is expressed as mean ± standard deviation (mean ± SD). Open-ended questions were analyzed separately, scored responses were summed and used in calculating the total scores of knowledge, attitudes, and perception for each participant. The knowledge of COVID-19 was assessed by answering 21 questions, then calculating each participant’s total cumulative knowledge score. Questions were given one point for the correct response and zero for the incorrect response. The association between knowledge score and sociodemographic variables was analyzed using the independent sample T-test and analysis variance (ANOVA). Post Hoc Analysis (LAD) was performed for multiple comparisons between every two categories. The statistical analysis was conducted at a 95% confidence level, and *p*-value < 0.05 was considered statistically significant. Statistical Package for the Social Sciences (SPSS) software, version 24 was used for statistical analysis.

## 3. Results

### 3.1. Baseline Characteristics of the Participants

Table 1 summarizes the baseline characteristics of the participants. Of the 389 participants, 256 (65.8%) were females, and 133 (34.2%) were males. Most participants (88.7%) were aged 19–24 years (88.7%), and some between 25 and 30 years (9.5 %), ≤18 years (1.5%), and >31 years (0.3%). Most participants lived in urban areas (80.7%), and some in rural areas (19.3%). The majority were medicine students (58.9%) and some pharmacy (15.2%), nursing (14.4%), and medical laboratory students (11.6%). The majority of participants were in the first (20.3%), second (27.5%), and third (20.6%) academic years.

### 3.2. Sources of Information about COVID-19

**Figure 1:**
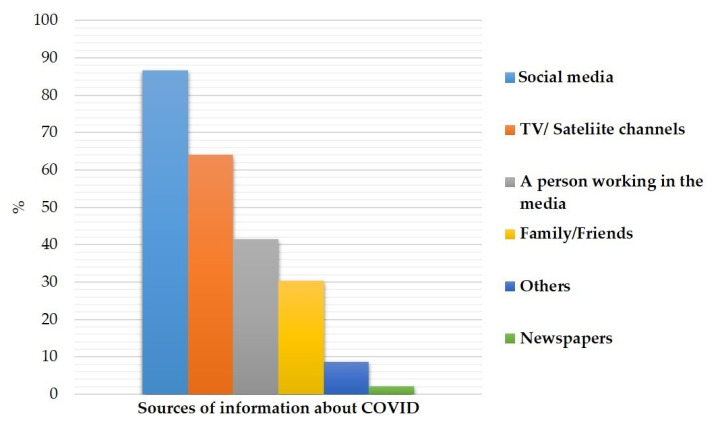
Sources of information about COVID-19.

Figure 1 summarizes sources of information about COVID-19. All participants claimed that they had heard about COVID-19. The majority of information sources were social media (86.6%), followed by TV/satellite channels (64%), people working in the media (41.4%), family/friends (30.3%), others (like World Health Organization, etc.) (2.1%), while 8.7% of information were from newspapers.

### 3.3. Knowledge of the Participants about Spread, Symptoms and Prevention of COVID-19

**Figure 2:**
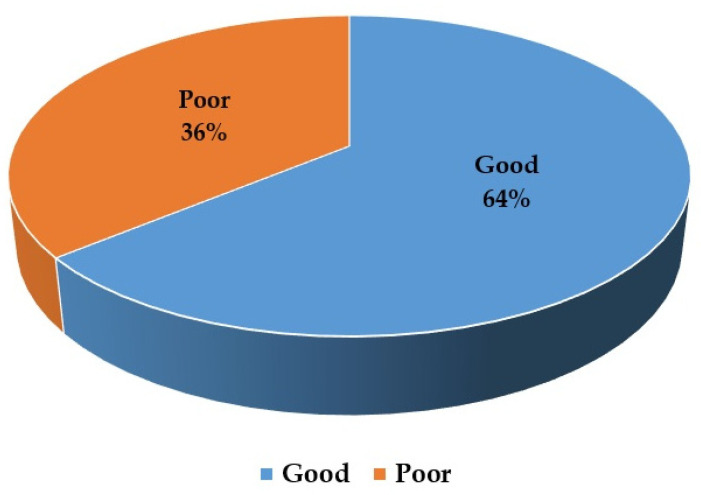
Knowledge level of the participants about COVID-19.

For each knowledge question, participants’ responses are presented in Table 2. Mean knowledge score was 16.17 ± 2.33 (Figure 2). Regarding COVID-19 transmission, the majority of participants (98.2%) agreed that nasal droplets are the main source of infection, then touching contaminated surfaces (92.5%), touching coins and banknotes (71.2%), asymptomatic people (71.2%), dealing with pets (19.0%), and stool contamination (15.2%). Regarding COVID-19 symptoms, difficulty breathing (99.5%), followed by fever (99.2%) and dry cough (97.9%) as the common symptoms of the disease, while body aches (71.7%), nasal congestion (59.4%), runny nose (46.0%), and diarrhea (35.0%) were the less common symptoms of the disease. The participants recognized the following as preventive measures for COVID-19: Proper hand hygiene (99.7%), maintaining an appropriate distance (99.2%), avoiding touching eyes and nose (98.2%), using face masks in public places (97.4%), taking antibiotics (9.3%), taking antiviral drugs (9.0%), and vaccine (1.5%). Moreover, 85.1% of participants agreed that the vaccine against the virus was unavailable, and 13.4% were not sure. Additionally, 73.5% were aware that antibiotics could not treat the virus, and 17.2% were not sure, while 19.3% had conflicting knowledge about antiviral drugs.

### 3.4. Attitudes of the Participants Preventive Measures against COVID-19

For each question focused on the attitudes of participants, the distribution of responses from participants is presented in Table 3. According to the survey results, 64.3% of participants strongly agree to wash their hands with soap and water, and 47.8% agree to use face masks to protect themselves from the risk of infection. Moreover, 43.2% of participants agree to inform the health authorities if they contacted a coronavirus-infected person, and 46.0% if they noticed any symptoms associated with the disease. Furthermore, 69.7% of participants reported to strongly agree to isolate themselves at home if they had contact with an infected person, and 41.9% strongly agreed to be isolated at the hospital until they prove that they are free from the disease. Additionally, 57.6% of participants would be willing to do the lab test if it’s available to detect the virus, 52.2% showed a willingness to get the vaccination if available, and 50.9% agreed to follow the updates about the spread of the virus in their country. In general, 52.7% of participants had positive attitudes towards protective measures against COVID-19.

### 3.5. Perception of the Participants about the Virus and the Disease

For each question focused on perception of participants, the distribution of responses from participants is presented in Table 4. According to the results, 59.6% of participants agreed that the virus is a dangerous disease, 72.8% were concerned about the possibility that they or their family members could get infected with the virus, but only 14.9% thought that the infection with COVID-19 is associated with stigma (for example, infected persons feel ashamed because people are afraid of and avoid them). Moreover, 64.5% of participants thought that the media coverage of this disease was exaggerated, and 34.2% thought that the virus was initially designed as a biological weapon.

### 3.6. Relationship between Sociodemographic of the Participants and Mean Knowledge Score about COVID-19

The relationship between the participants’ knowledge and their sociodemographic characteristics is shown in Table 5. The data show no statistically significant difference between mean knowledge scores of the participants living in urban and rural areas (16.27 ± 2.24 and 15.74 ± 2.64; *p* = 0.08), respectively. In contrast, there was a significant statistical difference between mean knowledge score of their gender (Table 5). Mean knowledge score was significantly higher in males than in females (16.52 ± 2.50 vs. 15.98 ± 2.21; *p* = 0.03), respectively. Moreover, there was a significant statistical difference between mean knowledge score of participants according to their specialty (Table 5). Furthermore, mean knowledge score was significantly higher in medicine students (16.59 ± 2.34) than students of medical laboratory (14.89 ± 2.26; *p* < 0.001) and nursing (15.67 ± 2.08; *p* = 0.008), respectively, while pharmacy students (16.00 ± 2.10) had a higher mean knowledge score than medical laboratory students (14.89 ± 2.26; *p* = 0.014). There was no significant difference between students of medicine and pharmacy (*p* = 0.077).

## 4. Discussion

The COVID-19 disease has caused enormous damage throughout the world. The World Health Organization (WHO) referred to this illness as the first coronavirus-caused pandemic on 11 March 2020. One of the largest nations in the Arab world is Yemen, with more than 35 million people. This large population may provide a significant spread and mortality risk, particularly for the elderly and those suffering from chronic conditions, including diabetes, hypertension, and heart disease. Global efforts have been exerted to prevent the spreading of the virus. These efforts include political efforts by the governments, together with the health workers, which depend on the awareness of the general public about the disease. Here we explore the results of our survey about KAP of the Hadhramout University Medical Students, the Corner Stone of the Health System. From the most recently available information, our work is the first survey that assessed the KAP of COVID-19 in Yemen.

In general, 64.0% of participants had a good knowledge score about the disease, its spread method, prevention, and treatment. This result is nearly close to studies among Egyptian (n = 283, knowledge score: 80.9%) [19], Jordanian (n = 592, knowledge score: 90%) [20], Turkish (n = 530, Knowledge score: 78%) [21], and Indian medical students (n = 1562, knowledge score: 71.2%) [22]. According to the information provided by the WHO, we divided the symptoms of the disease into common and less common ones and asked participants about these symptoms, which denoted excellent knowledge about this point. Our survey showed an excellent level of knowledge about the most common symptoms of the disease. These findings are in agreement with previous surveys on Egyptian [19] and Jordanian medical students [20].

Regarding information sources of COVID-19, the most important sources were social media (86.6%), while 8.7% of information was from international organizations (e.g., WHO). Social media (e.g., Facebook, WattsApp) plays an important role in virus protection by raising public awareness about protective measures and through countering rumors [23]. According to our survey, most participants used social media as their primary source of knowledge. Besides the current survey, previous studies emphasize the value of social media as a source of knowledge [23]. The improvement of the social media platform health system and visibility by better disseminating information to the public [24]. Yemeni Ministry of Health started using different means of communication, including television, mobile messages, as well as social media, including Facebook, to educate people about the disease.

On the other hand, most participants had good knowledge scores about the transmission of the virus through respiratory droplets and from infected persons to others, where 98.2% of participants agreed that the nasal droplets as the main source of infection and similar to other studies conducted to Egyptian (95%) [19] and Ugandan medical students (99%) [25] but it is less reported by the Pakistani students (70%) [26]. Moreover, 71.2% of the participants thought that coins could transmit the virus, and only 17.5% thought that asymptomatic people are not a part of the virus transmission chain. Regarding the disease symptoms, the survey revealed that breathing difficulties (99.5%), fever (99.2%), and dry cough (97.9%) were the most common symptoms of the disease and similar to an Egyptian study conducted by Abdelhafiz et al. [16]. This could be an important finding explained by various factors, such as the seriousness of the disease as circulated by different media and health authorities, especially after being declared as a pandemic by the WHO [27], and supported by the fact that most participants were aware of the common symptoms of the virus. For example, the majority of participants knew that fever, cough, and dyspnea could be the common clinical manifestation of COVID-19 [28]. The survey also showed high knowledge levels about disease preventive measures, such as regular proper hand hygiene (99.7%), maintaining an appropriate distance (99.2%), avoiding touching eyes and nose (98.7%), and using face masks in public places (97.4%), but less knowledge was related to disease treatment. Only less than ten percent of participants reported antibiotics and antiviral drugs as an option for preventing the disease. Furthermore, only 1.5% of participants reported them as a less common option. However, a Northern Thailand population study by Srichan et al. found that 31.2% were aware of the vaccine as a potential option [29].

Indeed, our survey found that more knowledge score was significantly higher in males than in females. Moreover, medicine students had more knowledge scores than students of medical laboratory and nursing, however, there was no significant difference between students of medicine and pharmacy. Similarly, Zhong et al. found that male sex, age group of 16–29 years, marital status, education, employment, and being a student were significantly associated with knowledge [30]. Therefore, tailoring the information provided by health officials and other media outlets on the disease needs to address the multifactorial nature of the drivers leading to reduced knowledge. Additionally, we discovered no discernible differences in students’ COVID-19 knowledge levels across urban and rural locations. However, Ferdous et al. discovered that young adults from rural areas had more accurate knowledge during the COVID-19 outbreak in Bangladesh, which may have been due to the fact that the majority of participants were students and that they all returned home, mostly to rural areas, during the lockdown period [16]. Preventive measures play a critical role in disease prevention and control. Our survey showed that about half of the participants (52.7%) reported a positive attitude score towards preventive measures against COVID-19, where 64.3% of participants stressed the value of regular hand washing, while 47.8% reported that putting on a facemask can protect from infection. These findings were also similar to a study conducted in China during the rapid rise period of the COVID-19 outbreak [29]. Saqlain et al. also reported positive attitudes among the vast majority of healthcare professionals towards wearing protective gear [31]. Saqlain et al. also reported that 80% of participants strongly agreed that COVID-19 transmission could be prevented by following universal precautions given by CDC or WHO [30]. During the SARS epidemic, 70.1–88.9% of Chinese residents believed that SARS can be successfully controlled or prevented [30,32]. Zhong et al. found that 90.8% of participants agreed with control measures, such as traffic limits throughout China and the shutdown of cities and counties of Hubei Province [30]. Furthermore, CDC and WHO recommended putting cloth face coverings for the public, especially in areas where there is significant community-based transmission [33]. On the other hand, WHO recommends using face masks only if a person has respiratory symptoms or caring for another person with symptoms [34]. Regarding self-isolation, our survey also found that 43.2% of participants strongly agreed to inform the health authorities if they contacted with an infected person and 46.0% if they had any symptoms associated with the disease. To minimize the crowd and slow the spread, the Yemeni government enforced a nighttime curfew for two weeks starting from the last week of April 2020. The decision included the closure of all restaurants, cafes, schools, and universities. At the same time, the government forced people to use facemasks in public places [10]. When we asked our participants about their perceptions regarding infection with the virus, most participants (59.6%) believed that it represents a life-threatening danger and were concerned about the potential risk of infection of any member of their families. Again, this reflects the effectiveness of the message provided by the different media platforms, which was confirmed by the negative assumptions that media is exaggerating the risk (28.8%). About 14.9% of participants thought the infection of the virus was associated with stigma. Although the number is limited, we think that it has significance, since it may lead to underreporting of cases, which may cause rapid spread of the disease. A cross-sectional survey conducted at one of the Egyptian university hospitals showed that healthcare workers had high levels of stigma towards people living with HIV [35]. We think the stigma towards COVID-19 is due to the fear of its mortality and high communicability, thus this issue can be resolved through continuous education and transparency of healthcare policies. Approximately 34.2% of participants thought that the virus started as a biological weapon. This limited number is interesting since it also reflects the growing awareness of the public. One year from the start of the COVID-19 pandemic in Yemen, stigma, fear of detention, and lack of knowledge about the presence of isolation centers continues to deter people from seeking timely treatment for the disease [35]. In Yemen, some patients stay at home for a while after they get symptoms and may arrive in the late stages of the disease [35]. Additionally, there are very few fully functional COVID-19 treatment centers. In other treatment centers, the health personnel often do not feel comfortable working without the required protective equipment, while fear of stigmatization hinders access to the few functional centers [35].

The major limitation of our work is that the survey was only distributed online, which allowed only students who had internet access to participate. Moreover, the sample was selected by convenience method. Moreover, our findings may not represent all Yemeni medical students.

## 5. Conclusions

Our survey found that Hadhramout University Medical Students had good knowledge about COVID-19, and a positive attitude toward using protective measures to limit the spread of infection. This knowledge is mainly acquired through social media platforms. However, there is an urgent need for scientific awareness campaigns regarding the disease. Meanwhile, healthcare authorities can use our survey as a baseline to evaluate more accurate knowledge among the Yemeni population towards COVID-19 or any pandemic in the future.

## Figures and Tables

**Table 1:** Baseline characteristics of the participants.

Participants No. (389)	No.	%
**Sex:**		
Male Female	133 256	34.2 65.8
**Age group (years):**		
≤18 19–24 25–30 30>	6 345 37 1	1.5 88.7 9.5 0.3
**Area of residence:**		
Rural area Urban urea	75 314	19.3 80.7
**Specialty:**		
Medicine Pharmacy Medical laboratory Nursing	229 59 45 56	58.9 15.2 11.6 14.4
**Academic level:**	79	20.3
First level Second level Third level Fourth level Fifth level Sixth level	107 80 61 40 22	27.5 20.6 15.7 10.3 5.7
Data are presented as frequencies (n) and percentage (%).		

**Table 2:** Knowledge of the participants about COVID-19.

Knowledge Items	Yes		No		Not Sure	
	No.	%	No.	%	No.	%
**COVID-19 spreads by:**						
1. Droplets of the affected person (with cough or expiration)	382	98.2	3	0.8	4	1.0
2. Surfaces touched by the affected person	360	92.5	3	0.8	21	5.4
3. Touching coins and banknotes	277	71.2	32	8.2	80	20.6
4. Dealing with pets	74	19	177	45.5	138	35.5
5. Stool (e.g., in public toilets)	59	15.2	207	53.2	123	31.6
6. The disease may spread from an asymptomatic person	277	71.2	68	17.5	44	11.3
**Symptoms of the disease:**						
7. Fever	386	99.2	0	0	3	0.8
8. Dry cough	381	97.9	3	0.8	5	1.3
9. Body aches	279	71.7	34	8.7	76	19.5
10. Runny nose	179	46.0	102	26.2	108	27.8
11. Diarrhea	136	35.0	143	36.8	110	28.3
12. Nasal congestion	231	59.4	71	18.3	87	22.4
13. Difficulty breathing	387	99.5	1.0	0.3	1.0	0.3
**Measures to prevent the viral spreading:**						
14. Proper hand hygiene	388	99.7	0	0	1	0.3
15. Maintaining an appropriate distance between yourself and anyone with symptoms	386	99.2	1	0.3	2	0.5
16. Avoiding touching eyes and nose	384	98.7	3	0.8	2	0.5
17. Putting on facemasks in public places	379	97.4	3	0.8	7	1.8
18. Taking antibiotics	36	9.3	286	73.5	67	17.2
19. Antiviral drugs can treat the virus	35	9.0	279	71.7	75	19.3
20. An effective vaccine against the virus when currently available	6	1.5	331	85.1	52	13.4
Mean knowledge score: 16.17 ± 2.33						
Data are presented as frequencies (n) and percentage (%).						

**Table 3:** Attitudes of the participants about COVID-19.

Attitude Items	Strongly Agree		Agree		Not Sure		Don’t Agree		Do Strongly Agree	
	No.	%	No.	%	No.	%	No.	%	No.	%
When I meet my friends and colleagues, I usually greet them with a handshake	48	12.3	67	17.2	17	4.4	119	30.6	138	35.5
2. When I meet my friends and colleagues, I usually great them with a hug	11	2.8	25	6.4	12	3.1	87	22.4	254	65.3
3. I wash my hands regularly and for enough period of time	250	64.3	26	6.7	4.1	16	53	13.6	44	11.3
4. I usually put on a facemask to protect myself from the risk of infection	110	28.3	76	19.5	29	7.5	113	29.0	61	15.7
5. If I find that I contacted a person infected with the virus, I will inform the health authorities	168	43.2	80	20.6	54	13.9	46	11.8	41	10.5
6. If I have any of the symptoms associated with the disease, I will inform the health authorities	179	46.0	76	19.5	40	10.3	40	10.3	54	13.9
7. If I find that I contacted a person infected with the virus, I agree to be isolated at home for a certain period of time until it is proven that I am free from the disease	271	69.7	46	11.8	24	6.2	4	1.0	44	11.3
8. If I found that I contacted a person infected with the virus, I agree to be isolated at an isolation hospital for a certain period of time until it is proven	163	41.9	68	17.5	41	10.5	47	12.1	70	18.0
9. If there is an available lab test for detection of the virus, I am willing to do it	224	57.6	76	19.5	31	8.0	19	4.9	39	10.0
10. If there is an available vaccine for the virus, I am willing to get it	203	52.2	78	20.1	40	10.3	23	5.9	45	11.6
11. I usually follow the updates about the spread of the virus in my country	198	50.9	63	16.2	31	8.0	57	14.7	40	10.3
12. I usually follow the updates about the spread of the virus worldwide	132	33.9	88	22.6	43	11.1	85	21.9	41	10.5
13. If a lecture about the virus is organized near me, I am willing to attend it	127	32.6	70	18.0	45	11.6	79	20.3	68	17.5
14. If flyers or brochures that include information about the disease are distributed, I will read them and follow the instructions mentioned	204	52.4	89	22.9	40	10.3	22	5.7	34	8.7
15. If protective measures and equipment are available at an affordable price, I will buy them	261	67.1	55	14.1	22	5.7	7	1.8	44	11.3
Data are presented as frequencies (n) and percentage (%).										

**Table 4:** Perception of the participants about COVID-19.

Perceptions Items	Yes		No		Not Sure	
	No.	%	No.	%	No.	%
I think that this disease is dangerous	232	59.6	136	35	21	5.4
2. I am concerned about the possibility that I or another family member will get infected with this virus	283	72.8	91	23.4	15	3.9
3. Infection with the virus is associated with stigma (for example, infected persons feel ashamed because people are afraid of and avoid them)	58	14.9	315	81	16	4.1
4. I think the media coverage of this disease is exaggerated	251	64.5	112	28.8	26	6.7
5. I think this virus was initially designed as a biological weapon	133	34.2	96	24.6	160	41.1
Data are presented as frequencies (n) and percentage (%)						

**Table 5:** Relationship between sociodemographic of the participants and their mean knowledge score about COVID-19.

Sociodemographic Data	Mean Knowledge Score		*p*-Value
	Min-Max	Mean ± SD	
Sex Male Female	5–22 10–22	16.52 ± 2.50 15.98 ± 2.21	0.029
Residence area Urban Rural	5–20 10–22	16.27 ± 2.24 15.75 ± 2.64	0.080
Specialty Medicine Pharmacy Medical laboratory Nursing	5–22 11–19 10–19 10–21	16.58 ± 2.34 16.00 ± 2.10 14.89 ± 2.25 *^,†^ 15.67 ± 2.08 *	<0.001

Data are represented by mean ± standard deviation (mean ± SD). Independent sample *t*-test was used to compare the mean values knowledge score of the participants according to sex and residence area. Analysis of Variance (ANOVA) was done. Post Hoc Analysis (LAD) was performed for multiple comparisons between every two groups.* represents medical laboratory and nursing groups and are significantly different medicine groups, ^†^ represents medical laboratory group is significantly different pharmacy group. Statistical analysis was done at a 95% confidence interval, and the differences were considered statistically significant if *p* < 0.05.

## Data Availability

Data are available from the corresponding author upon request.
